# Optimization of Medium Composition for Biomass Production of *Lactobacillus plantarum* 200655 Using Response Surface Methodology

**DOI:** 10.4014/jmb.2103.03018

**Published:** 2021-03-26

**Authors:** Ga-Hyun Choi, Na-Kyoung Lee, Hyun-Dong Paik

**Affiliations:** Department of Food Science and Biotechnology of Animal Resources, Konkuk University, Seoul 05029, Republic of Korea

**Keywords:** Probiotics, *Lactobacillus plantarum*, medium optimization, plackett-burman design, response surface methodology

## Abstract

This study aimed to optimize medium composition and culture conditions for enhancing the biomass of *Lactobacillus plantarum* 200655 using statistical methods. The one-factor-at-a-time (OFAT) method was used to screen the six carbon sources (glucose, sucrose, maltose, fructose, lactose, and galactose) and six nitrogen sources (peptone, tryptone, soytone, yeast extract, beef extract, and malt extract). Based on the OFAT results, six factors were selected for the Plackett- Burman design (PBD) to evaluate whether the variables had significant effects on the biomass. Maltose, yeast extract, and soytone were assessed as critical factors and therefore applied to response surface methodology (RSM). The optimal medium composition by RSM was composed of 31.29 g/l maltose, 30.27 g/l yeast extract, 39.43 g/l soytone, 5 g/l sodium acetate, 2 g/l K_2_HPO_4_, 1 g/l Tween 80, 0.1 g/l MgSO_4_·7H_2_O, and 0.05 g/l MnSO_4_·H_2_O, and the maximum biomass was predicted to be 3.951 g/l. Under the optimized medium, the biomass of *L. plantarum* 200655 was 3.845 g/l, which was similar to the predicted value and 1.58-fold higher than that of the unoptimized medium (2.429 g/l). Furthermore, the biomass increased to 4.505 g/l under optimized cultivation conditions. For lab-scale bioreactor validation, batch fermentation was conducted with a 5-L bioreactor containing 3.5 L of optimized medium. As a result, the highest yield of biomass (5.866 g/l) was obtained after 18 h of incubation at 30°C, pH 6.5, and 200 rpm. In conclusion, mass production by *L. plantarum* 200655 could be enhanced to obtain higher yields than that in MRS medium

## Introduction

The consumption of probiotics as functional foods or supplements has increased with the growing interest of consumers. Due to various health benefits such as balancing microbiota composition, improving immunity, protecting against intestinal pathogens, and controlling bowel diseases, the popularity of probiotics has grown remarkably in recent years [1]. As the importance of probiotics is consistently emphasized, research related to large-scale industrial production of probiotics has become necessary to satisfy the increasing demand.

Lactic acid bacteria (LAB), including the genera *Bifidobacterium*, *Lactobacillus*, *Lactococcus*, *Enterococcus*, and *Streptococcus*, are normally regarded as probiotics [1]. Probiotic LAB can survive in the human gut and play a vital role not only in the gut microbiome but also in the brain. They offer therapeutic effects against various diseases by producing bioactive molecules such as short-chain fatty acids, γ-aminobutyric acid, exopolysaccharides, acetylcholine, serotonin, and vitamins [2]. Additionally, probiotics show anti-allergic, antioxidant, immunostimulatory, and cholesterol-lowering effects [3-5].

Among the strains of lactobacilli, which consist of more than 50 species, *Lactobacillus plantarum* is one of the most widespread, versatile LAB traditionally used in the fermentation of vegetables, meat, and dairy [6]. *L. plantarum* 200655, which was used in this study, was isolated from Korean kimchi. This strain exerts antioxidant effects and can enhance immunity by stimulating the production of cytokines such as IL-1β and IL-6 [7]. Moreover, heat-killed *L. plantarum* 200655 shows neuroprotective effects against oxidative stress induced by H_2_O_2_ [8].

The commercial medium used for *Lactobacillus* is MRS (de Man, Rogosa, and Sharp), which is comprised of essential nutrients (acetate, magnesium, manganese, and polysorbate) and growth-inhibiting ingredients to prevent undesirable bacterial growth. However, the MRS medium is insufficient for maximizing the growth of some *Lactobacillus* strains [9]. Lactobacilli are fastidious bacteria that require rich media because their capacity to metabolize nutrients, such as sugars, amino acids, peptides, and vitamins, varies depending on the strain [6, 10]. Therefore, the production of viable cells, biomass, and metabolites of *L. plantarum* is significantly affected by the components of the medium and cultivation conditions. If a new medium for each strain or species could be devised as an alternative to conventional media, it would lead to enhanced biomass production and economical benefits including the reduction of cost, wastewater, and fermentation time [10, 11].

To find effective ways to increase the yield of desired products, we optimized the medium using various strategies. One of the classical methods is one-factor-at-a-time (OFAT) in which only one factor is changed while the other factors are fixed constant. Although it is simple and convenient, OFAT involves a large number of experiments with an increasing number of factors. Moreover, the method disregards the interactions between factors [12, 13]. Thus, statistical methods such as Plackett-Burman design (PBD), factorial design, and response surface methodology (RSM) are preferred owing to their efficiency. Furthermore, RSM includes factorial designs and multiple regression analysis and considers the effects of factors, relationships between variables, and optimal conditions [14].

Although many studies have reported medium optimization of *L. plantarum* to enhance its production of metabolites such as lactic acid [14, 15], bacteriocin [16], and exopolysaccharides [17, 18], few studies have focused on biomass production by *L. plantarum* [11, 19]. Therefore, we used RSM to optimize the medium and fermentation conditions to enable enhanced biomass production by *L. plantarum* 200655.

## Materials and Methods

### Bacterial Strain and Medium

*L. plantarum* 200655 (KCCM 12204P) isolated from Korean kimchi was used in this study. Stock cultures were stored at -80°C in MRS broth (Difco, USA) with 20% (v/v) sterile glycerol. The strain was incubated at 37°C for 24 h in MRS medium and activated at least twice before use.

The basal medium was derived from MRS medium and consisted of 5 g/l sodium acetate (Sigma, USA), 2 g/l K_2_HPO_4_ (Samchun, Korea), 1 g/l Tween 80 (Yakuri Pure Chemicals Co. Ltd., Japan), 0.1 g/l MgSO_4_·7H_2_O (Shinyo Pure Chemicals Co., Japan), and 0.05 g/l MnSO_4_·H_2_O (Samchun). Basal medium was added to all media used in this study. The initial pH of all media was adjusted to 6.5 ± 0.05 with 1 M NaOH and 1 M HCl before sterilization. The MRS medium was used as a control (unoptimized medium) for comparison with the optimized medium.

Each fermentation condition for optimized medium was as follows: A colony was inoculated in MRS broth and incubated to an optical density (OD) of 0.5 ± 0.05 at 600 nm. One hundred milliliters of the medium were inoculated with 1% (v/v) of seed culture in a 250-ml Erlenmeyer flask and incubated at 37°C for 24 h without shaking.

### Measurement of Biomass

Culture broth (50 ml) was collected and separated by centrifugation at 4,000 ×*g* for 15 min at 4°C. The supernatant was discarded, and the cell pellets were washed twice with sterile distilled water. Biomass was obtained by drying at 80°C until it reached a constant weight in a dry oven.

### Determination of Medium Compositions Using OFAT

To investigate the effects of carbon and nitrogen sources on bacterial cell mass, the OFAT method was used. This method can estimate the effects of each variable, but it does not consider the interactions between variables. Before proceeding with the OFAT method, the API 50 CHL Medium Kit (BioMerieux, France) was used to assess the availability of metabolizing carbohydrates. Glucose, sucrose, maltose, fructose, lactose, and galactose were selected, and six carbon sources were added individually to 100 ml of basal medium containing 10 g/l yeast extract at a concentration of 20 g/l. Likewise, six nitrogen sources (peptone, soytone, tryptone, yeast extract, beef extract, and malt extract) were added to 100 ml of basal medium containing 20 g/l glucose at a concentration of 10 g/l.

### Plackett-Burman Design (PBD)

PBD was used to screen for significant factors affecting the biomass production by *L. plantarum* 200655. Based on the preliminary screening results, three highly influential variables were selected for each of the carbon and nitrogen sources. Those six variables were maltose, sucrose, lactose, yeast extract, soytone, and tryptone, and the variables represented at high and low levels were coded as +1 and -1, respectively. The design matrix with 12 runs and the actual values of the variables are shown in Table 2. The response values indicate the biomass of *L. plantarum* 200655. All experiments were conducted in triplicate. The variables that had positive effects on biomass production at a confidence level higher than 95% were considered for further experiments.

### Central Composite Design and Response Surface Methodology

The RSM with CCD was conducted to optimize the concentration of medium components and estimate the effects of each variable and the interactions between variables. Maltose, yeast extract, and soytone were used as the independent variables. Variables were set at five different levels (-α, -1, 0, 1, α), and the range of actual values is presented in Table 4. The experimental design of the CCD was made up of a full factorial design with six center points (Table 5). The media were prepared according to a combination of variables in the experimental runs, and all experiments were conducted under static conditions. The obtained biomass was used to establish the regression model, and the quadratic polynomial equation for the variables was as follows:

$$Y=\beta_{{}_{0}}+\sum \beta_{i}X_{i}+\sum \beta_{ij}X_{i}X_{j}+\sum \beta_{ii}X_{i}^{2}$$

where Y is the response value of the dependent variable (biomass of *L. plantarum* 200655); β_0_, β_i_, β_ij_, and β_ii_ are intercept, linear, interaction, and squared coefficients of the model, respectively; Xi and Xj are the independent variables.

### Effects of Culture pH and Temperature

The effects of initial pH and incubation temperature were investigated to determine the optimal cultivation conditions for *L. plantarum* 200655. The pH was adjusted from 4.0 to 9.0 with 1 M NaOH and 1 M HCl before sterilization, and the incubation temperature was set from 22 to 37°C. The optimal conditions were assessed by comparing the biomass after 24 h of incubation.

### Scale-Up Fermentation of Optimized Medium

Scale-up fermentation of *L. plantarum* 200655 under the optimized medium and conditions was performed in a 5-l bioreactor with a working volume of 3.5 l. The medium was inoculated with 1% (v/v) of seed culture grown in the MRS medium for 24 h. The temperature was controlled at 30°C, and the pH was maintained at 6.5, with 3 M NaOH and 3 M HCl. The agitation speed was maintained at 200 rpm. The MRS medium was used as a control, and fermentation was performed under the same conditions. Biomass and cell viability were measured for 24 h. Fifty milliliters of culture broth was collected to measure the biomass, and cell viability was measured on MRS agar plates after incubation at 37°C for 24 h.

### Statistical Analysis

All experiments were repeated in triplicate. Data are presented as the mean ± SD. One-way analysis of variance and Duncan’s multiple range test were used to determine the degree of significant differences. Values were considered significant at *p* < 0.05, and all analyses were performed using SPSS (IBM, USA). PBD and RSM were performed using Minitab 18 software (Minitab 18 Inc., USA).

## Results and Discussions

### Effects of Carbon and Nitrogen Sources on Biomass Production by *L. plantarum* 200655

Various carbon and nitrogen sources were evaluated to identify the factors that have profound effects on biomass production by *L. plantarum* 200655. The basal medium contained 20 g/l of each carbon source and 10 g/l of each nitrogen source. The biomass obtained from maltose was 2.253 g/l, which was the highest value among the six carbon sources, whereas sucrose, lactose, glucose, fructose, and galactose were 1.753, 1.749, 1.703, 1.459, and 1.401 g/l, respectively. Maltose was the most preferred carbon source by *L. plantarum* 200655, compared to other carbon sources (*p* < 0.05).

Maltose utilization is related to maltose-related genes such as MALS (encoding maltase gene), MALT (encoding maltose permease gene), and MALR (transcriptional activator gene of the MALS and MALT). These results are similar to those of a study by Yeo *et al*. [9], where maltose was determined to be a suitable carbon source for *Lactobacillus salivarius* W13 among eight carbon sources. Maltose medium showed significant cell growth of 5.2 × 10^8^ CFU/ml, which was 2.47-fold higher than that in MRS medium. Lim *et al*. [20] reported that the cell growth by *Lactobacillus brevis* HYE1 increased proportionally in accordance with maltose concentrations between 0.2 to 4%. Although maltose was an optimal carbon source for *L. plantarum* 200655, the three highest components (maltose, sucrose, and lactose) were selected for the following steps.

Table 1 also shows the biomass according to the six nitrogen sources. It was obvious that different nitrogen sources in the medium had different effects on biomass production. When malt extract was added to the medium, *L. plantarum* 200655 showed a tremendously low biomass production (0.022 g/l). Although malt extract could help *L. plantarum*, *L. acidophilus*, and *L. reuteri* survive in acidic conditions at pH 2.5 [21], *L. plantarum* 200655 cannot utilize the malt extract as a nitrogen source for growth. When 2.5% malt extract was added to the medium, *L. brevis* HYE1 showed similar levels of cell growth to that in the absence of any nitrogen source [20]. Beef extract is also not effective for the growth of *L. plantarum* 200655. Yeast extract resulted in maximum biomass production (1.722 g/l), followed by soytone (1.480 g/l), and tryptone (1.371 g/l). When 50% yeast extract in the medium was replaced with beef or malt extract, the biomass of *Lactobacillus sakei* CCUG 42687 was reduced from 3.9 to 3.6 g/l and then to 2.7 g/l [22]. According to Lee *et al*. [24], the highest cell density of *L. acidophilus* A12 was obtained in yeast extract medium, whereas the strain showed little preference for beef and malt extracts. Yeast extract promoted high biomass production and lactic acid metabolism in LAB owing to its higher abundance of nitrogenous bases and vitamin B [15].

### Plackett-Burman Design for Screening Variables

**Fig. 1:**
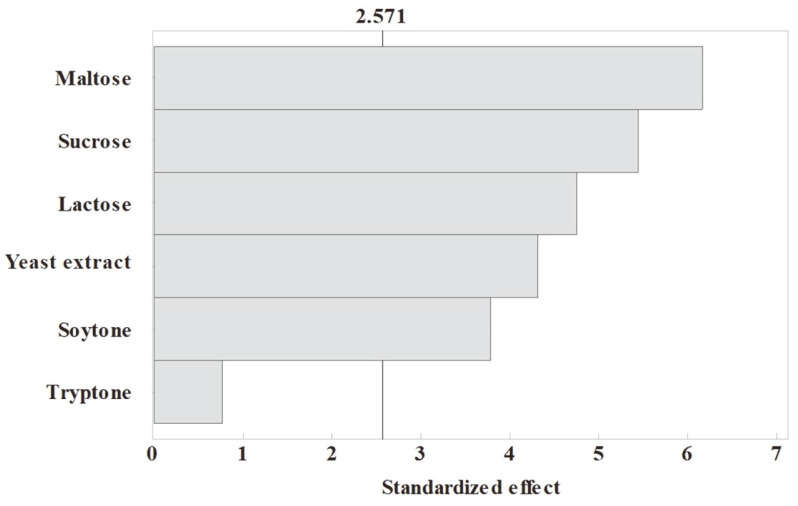
Pareto chart depicting the standard effects of six factors on the biomass production of *L. plantarum* 200655.

Because PBD is an efficient tool for estimating the main effect of each variable, it was applied to identify the most significant variables before performing RSM. Maltose, sucrose, lactose, yeast extract, soytone, and tryptone were selected as variables for PBD. They were set at two different levels (-1, 1), where the actual values were 10 to 30 g/l and 5 to 10 g/l respectively. The six variables with 12 experimental runs resulted in biomass production ranging from 2.036 to 2.561 g/l (Table 2). The experiments were conducted in triplicate in accordance with combinations of variables. The standardized effects of the variables are represented as a single column on the Pareto chart (Fig. 1). The vertical line through the column indicates whether the variables are statistically significant. Where the columns crosses over the line and extends to the right shows that the variables had a large effect on the biomass. Table 3 shows the effects of the variables and analysis of variance. The effects of maltose (X_1_), sucrose (X_2_), lactose (X_3_), yeast extract (X_4_), soytone (X_5_), and tryptone (X_6_) were 0.1884, 0.0236, -0.1318, 0.1667, 0.1456, and 0.1158, respectively. Only lactose had a negative effect on biomass production among the six variables, suggesting that biomass production was decreased by increasing the concentration of lactose in the medium in the tested concentration of 10 g/l to 30 g/l. In contrast, maltose had the highest effect, followed by the yeast extract and soytone. The analysis of variance showed that all components except sucrose were statistically significant (*p* < 0.05). Even though tryptone had positive effects and low probability value, it was excluded from further optimization process because the other two nitrogen sources had higher effects than tryptone. Thus, maltose, yeast extract, and soytone were selected as the most important factors for RSM. According to Aasen *et al*. [22], the best production of biomass and bacteriocin by *L. sakei* CCUG 42687 was obtained when the medium contained yeast extract and soytone at the same ratio. The model acquired from PBD was fitted well because the determination coefficient (R^2^) was 0.9612, which indicated that the model could explain 96.12% of its variability.

### Optimization of Medium Components Using Response Surface Methodology

The CCD model was designed based on RSM to determine the optimal concentrations of maltose (X_1_, 8.18 g/l to 41.8 g/l), yeast extract (X_2_, 8.18 g/l to 41.8 g/l), and soytone (X_3_, 4.77 g/l to 55.2 g/l). The coded units and actual concentrations of the three independent variables are listed in Table 4. Table 5 shows the design matrix consisting of 20 runs and experimental responses, where biomass production varied from 2.348 to 3.892 g/l. The highest biomass was obtained in run 8 with a concentration of 35 g/l maltose, 35 g/l yeast extract, and 45 g/l soytone. The responses of biomass were analyzed by applying multiple regression analysis, and the second-order polynomial equation, which expressed the relationship between the predicted response and variables, was as follows:

$$Y=3.7475+0.3784X_{1}+0.1012X_{2}+0.1772\text{​}X_{3}-0.2922X_{1}^{2}-0.1002\text{​}X_{2}^{2}-0.1402X_{3}^{2}+0.0015X_{1}X_{2}-0.0105X_{1}X_{3}+0.0090X_{2}X_{3}$$

where X_1_, X_2_, and X_3_ are maltose, yeast extract, and soytone, respectively.

The analysis of variance presented in Table 6 shows the significance of the quadratic regression model with linear, squared, and interaction terms. As shown in Table 6, a high *F*-value (33.22) with a very low probability value ((*p* > F) < 0.0001) was obtained. The *F*-value is the ratio of the mean square regression to the mean square residual. The critical value of F _(8, 10, 0.05)_ tabulated on the *F* distribution table was 3.07. The calculated *F*-value greatly exceeded the critical value of *F*, meaning that the null hypothesis could be rejected and that the model was highly significant. The coefficient of determination (R^2^) that explains the fit of the model was 0.9676, implying that 96.76% of the total variation in response could be elucidated, and only 3.24% of the variability was not explained by the model. Usually, the R^2^ value exists between 0 and 1, and at values closer to 1, the model could predict the response better. The model with R^2^ > 0.75 is regarded as acceptable [24]. Moreover, the adjusted coefficient of determination (R^2^ = 0.9385) and multiple correlation coefficient (R = 0.9837) also showed high values, which advocated the close correspondence between the empirical and predicted values. Additionally, the accuracy of the model was also demonstrated by a statistically insignificant lack of fit (*p* > 0.05). The lack of fit indicates that the model failed to express data in the experimental domain at points not involved in the regression [20]. The linear and quadratic effects of maltose (X_1_), yeast extract (X_2_), and soytone (X_3_) significantly affected the biomass of *L. plantarum* 200655 (*p* < 0.05). Maltose was the most significant factor (*p* < 0.0001), followed by soytone (*p* = 0.0002) and yeast extract (*p* = 0.0089). Maltose, yeast extract, and soytone can function as limiting nutrients because the quadratic effects of the three variables are highly significant (*p* < 0.05), meaning that a slight modification of their concentrations can affect the biomass [25].

**Fig. 2:**
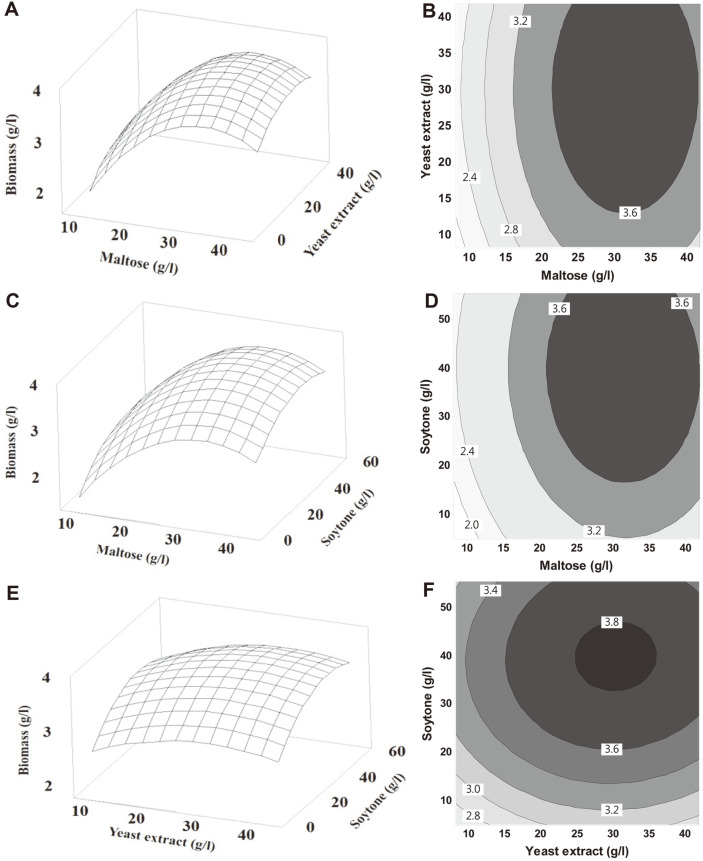
Response surface plots and contour plots for biomass production of *L. plantarum* 200655. (**A, B**) Interaction between maltose (X_1_, g/l) and yeast extract (X_2_, g/l) with soytone (X_3_, g/l) at zero level. (**C, D**) Interaction between maltose (X_1_, g/l) and soytone (X_3_, g/l) with yeast extract (X_2_, g/l) at zero level. (**E, F**) Interaction between yeast extract (X_2_, g/l) and soytone (X_3_, g/l) with maltose (X_1_, g/l) at zero level.

Three-dimensional response surface and contour plots were drawn to express the interactions between the two variables and derive the optimal concentration for maximal biomass production (Fig. 2). Graphical representations having a convex-shaped response surface were depicted based on the model equation. Each graph showed the infinite combinations of two independent variables, with the other one at a constant level. In the optimization of the medium for maximal actinorhodin production by *Streptomyces coelicolor* A3(2) using RSM, no interactions were found between variables (X_1_X_2_, X_1_X_3_, and X_2_X_3_) because contour plots were relatively round in nature [25]. The insignificance of the interaction terms was also supported by a high probability value (*p* > 0.05). As shown in Figs. 2A and 2B, biomass increased at a maltose concentration between 8.18 g/l and 31.29 g/l, and it declined at concentrations beyond 31.29 g/l. The concentration of yeast extract and soytone were also analyzed, while that of maltose was maintained at the central point. When the concentration of yeast extract reached 30.27 g/l, a peak in biomass production was observed. In the case of soytone, a similar profile was observed; the biomass increased at soytone concentrations up to 39.43 g/l. It seems that high concentrations of the independent variables suppressed the increase in biomass production. When the concentrations of maltose, yeast extract, and soytone were 31.29 g/l, 30.27 g/l, and 39.43 g/l, respectively, the regression model predicted a maximum biomass concentration of 3.951 g/l, with a 95% confidence interval ranging between 3.835 and 4.067 g/l.

The predicted model was validated under the optimized conditions by performing independent experiments in triplicate, and the biomass was found to be 3.845 g/l. The model showed good agreement with the predicted value, and the experimental value was within the 95% confidence interval range. The biomass in the optimized medium was 1.58-fold higher compared to that in the unoptimized medium (2.429 g/l). Manzoor *et al*. [11] developed a low-cost medium consisting of 15 g/l glucose, 60 g/l cheese whey, and 15 g/l corn steep liquor using the Box-Behnken method. Viable cells and dry cell mass of *L. plantarum* AS-14 were significantly increased (*p* < 0.05) when incubated in the optimized medium. Selvamani *et al*. [24] used RSM to optimize a medium containing 68.60 g/l lactose, 50 g/l yeast extract, and 1 g/l K_2_HPO_4_, which resulted in maximum biomass production (3.96 g/l) by *L. reuteri* DSM20016^T^. Moreover, the optimum concentrations of the medium components for *L. plantarum* ATCC 8014 were 40 g/l molasses, 16.8 g/l yeast extract, 2.72 g/l KH2PO_4_, and 3.98 g/l sodium acetate. After 48 h of fermentation in the optimized medium, a cell mass of 4.40 g/l was achieved, which increased up to 4.5 times compared to that in the unoptimized medium [18].

### Effects of pH of Medium and Fermentation Temperature on Biomass

**Fig. 3:**
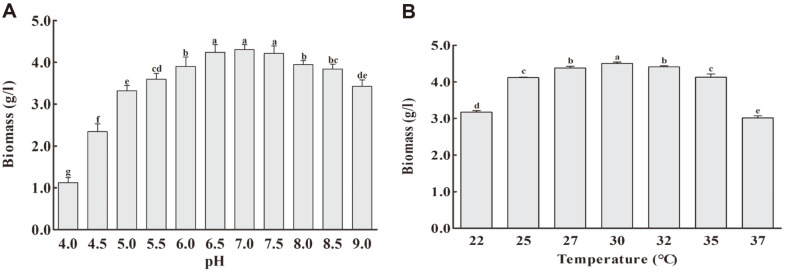
Effects of initial pH (A) and incubation temperature (B) on biomass production by *L. plantarum* 200655. Data are presented as the mean ± SD of independent experiments in triplicate. Different superscript letters of each figure are significantly different (*p* < 0.05).

The effects of the initial pH and incubation temperature were investigated in the optimized medium since not only the medium formula but also the physicochemical parameters are important for bacterial growth. It seems that the pH and temperature affected the biomass production by *L. plantarum* 200655. As shown in Fig. 3A, the highest biomass (4.304 g/l) was obtained at pH 7.0, which decreased sequentially above and below this point. The lowest biomass (1.124 g/l) was obtained when the pH was 4.0. The biomass decreased dramatically from pH 4.0 to 5.0, whereas the decline from pH 8.0 to 9.0 was gradual. Low pH was found to inhibit the growth of *L. plantarum* 200655. Even though the optimal pH for most *Lactobacillus* strains was suggested to be between 5.0 to 6.0, low pH conditions (< 4.4) could inhibit the growth and slow down the growth rate [26]. Moreover, it is possible to grow some strains of LAB under alkaline conditions by regulating the internal pH by alkalization of the cytoplasm [27]. While acidic and alkaline culture conditions negatively influenced cell growth, neutral conditions were favorable to *L. plantarum* 200655. Although there were little differences in biomass production within the pH range of 6.5 to 7.5, the biomass at pH 7.0 was insignificantly different from that at pH 6.5 (*p* = 1.000) and pH 7.5 (*p* = 0.999). Therefore, pH 6.5 was chosen as the optimal pH for subsequent experiments.

The effects of temperature were also evaluated using the optimized medium. The maximal biomass (4.505 g/l) was attained at 30°C, which was statistically significant compared to that at other temperatures (*p* < 0.05). The biomass decreased when temperatures exceeded 30°C, and the lowest biomass (3.019 g/l) was observed at 37°C. Optimal biomass production was observed within the pH range of 6.5 to 7.0 and a temperature of 30°C. According to Manzoor *et al*. [11], the best conditions for maximum biomass production by *L. plantarum* AS-14 were a temperature of 34–39.6°C and a pH of 6.1–6.4. Some studies have reported that the maximum biomass production by LAB was obtained at pH ranging from 5.2 to 6.3 and an incubation temperature ranging from 22 to 40°C [10, 11, 16, 28, 29].

### Comparison of Optimized and Unoptimized Media in a Bioreactor

**Fig. 4:**
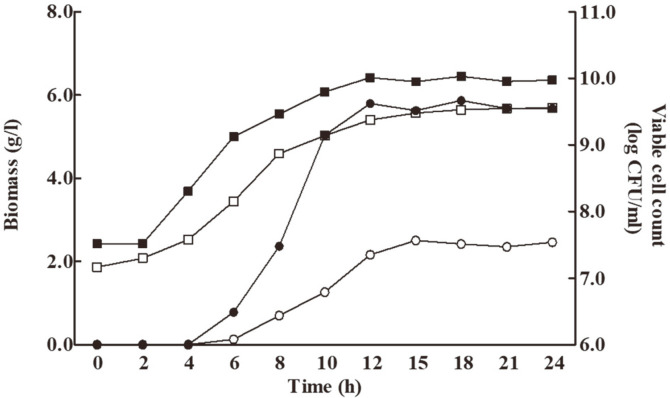
The time course of biomass production and viable cells of *L. plantarum* 200655 in optimized medium (●, ■) and unoptimized medium (○, □).

Fig. 4 presents the time course of *L. plantarum* 200655 regarding biomass production and viable cells cultivated in optimized and unoptimized media (MRS) under the same incubation conditions (30°C, pH 6.5, and an agitation speed of 200 rpm). The fermentation was performed for 24 h in a 5-l bioreactor containing 3.5 l of each medium. Although a readable increase in biomass was not observed from 0 to 4 h of incubation in both media, the biomass increased dramatically at 6 h of fermentation. The highest biomass was obtained after 18 and 15 h of fermentation in the optimized medium (5.866 g/l) and MRS medium (2.504 g/l), respectively. A significant difference in biomass production (2.34-fold increase) was observed. The viable cells of *L. plantarum* 200655 are also represented in Fig. 4. Although the maximum number of viable cells (9.56 log CFU/ml) was observed in the MRS medium after 24 h of incubation, a higher number of viable cells (10.03 log CFU/ml) was observed in the optimized medium at a shorter fermentation time of 18 h. It seems that the optimized medium affected not only the biomass but also the number of viable cells of *L. plantarum* 200655. Selvamani *et al*. [24] investigated the growth kinetics of the dry cell weight of *L. reuteri* DSM 20016^T^, and the maximum cell mass was observed after 48 h of fermentation in an optimized medium by RSM. The biomass of *L. reuteri* DSM 20016^T^ increased 3-fold (3.96 g/l) in the optimized medium compared to the unoptimized medium (1.76 g/l). Fonteles *et al*. [29] reported that *Lactobacillus casei* B-442 showed the highest number of viable cells (8.93 log CFU/ml) in fermented cantaloupe juice under optimized conditions (fermentation temperature at 31°C, initial pH 6.1 for 24 h).

Therefore, biomass production by *L. plantarum* 200655 could be optimized in a medium comprising 31.29 g/l maltose, 30.27 g/l yeast extract, 39.43 g/l soytone, 5 g/l sodium acetate, 2 g/l K_2_HPO_4_, 1 g/l Tween 80, 0.1 g/l MgSO_4_·7H_2_O, and 0.05 g/l MnSO_4_·H_2_O under the culture conditions of 30°C, pH 6.5, and an agitation speed of 200 rpm. In conclusion, this study verified the statistically-optimized medium composition and culture conditions that could be used for enhanced biomass production by *L. plantarum* 200655.

## Figures and Tables

**Table 1:** Effects of various carbon and nitrogen sources on biomass production of *L. plantarum* 200655.

Components		Biomass (g/l)
Carbon sources	Glucose	1.703
	Sucrose	1.753
	Maltose	2.253
	Fructose	1.459
	Lactose	1.749
	Galactose	1.401
Nitrogen sources	Peptone	1.175
	Soytone	1.480
	Tryptone	1.371
	Yeast extract	1.722
	Beef extract	0.495
	Malt extract	0.022

**Table 2:** Experimental design and response values of Plackett-Burman design.

Run	Variables^a^						Biomass (g/l)

	X_1_ (g/l)	X_2_ (g/l)	X_3_ (g/l)	X_4_ (g/l)	X_5_ (g/l)	X_6_ (g/l)	
1	1 (30)	-1 (10)	1 (30)	-1 (5)	-1 (5)	-1 (5)	2.036
2	1 (30)	1 (30)	-1 (10)	1 (10)	-1 (5)	-1 (5)	2.407
3	-1 (10)	1 (30)	1 (30)	-1 (5)	1 (10)	-1 (5)	2.109
4	1 (30)	-1 (10)	1 (30)	1 (10)	-1 (5)	1 (10)	2.407
5	1 (30)	1 (30)	-1 (10)	1 (10)	1 (10)	-1 (5)	2.561
6	1 (30)	1 (30)	1 (30)	-1 (5)	1 (10)	1 (10)	2.360
7	-1 (10)	1 (30)	1 (30)	1 (10)	-1 (5)	1 (10)	2.232
8	-1 (10)	-1 (10)	1 (30)	1 (10)	1 (10)	-1 (5)	2.181
9	-1 (10)	-1 (10)	-1 (10)	1 (10)	1 (10)	1 (10)	2.432
10	1 (30)	-1 (10)	-1 (10)	-1 (5)	1 (10)	1 (10)	2.514
11	-1 (10)	1 (30)	-1 (10)	-1 (5)	-1 (5)	1 (10)	2.122
12	-1 (10)	-1 (10)	-1 (10)	-1 (5)	-1 (5)	-1 (5)	2.079

Actual values are presented in parentheses.

^a^X_1_, maltose; X_2_, sucrose; X_3_, lactose; X_4_, yeast extract; X_5_, soytone; X_6_, tryptone.

**Table 3:** Analysis of variables based on Plackett-Burman design.

Variables	Effect	Coefficient	*T*-value	*P*-value
Intercept		2.2867	149.56	< 0.0001
X_1_	0.1884	0.0942	6.16	0.0016
X_2_	0.0236	0.0118	0.77	0.4759
X_3_	-0.1318	-0.0659	-4.31	0.0076
X_4_	0.1667	0.0833	5.45	0.0028
X_5_	0.1456	0.0728	4.76	0.0051
X_6_	0.1158	0.0579	3.79	0.0128

**Table 4:** Coded and real values of independent variables used in the central composite design.

Independent variables (g/l)	Actual levels of coded values				

	-α	-1	0	1	α
Maltose (X_1_)	8.18	15	25	35	41.8
Yeast extract (X_2_)	8.18	15	25	35	41.8
Soytone (X_3_)	4.77	15	30	45	55.2

**Table 5:** Central composite design and response values.

Run	Independent variables^a^			Biomass (g/l)

	X_1_	X_2_	X_3_	
1	-1	-1	-1	2.484
2	1	-1	-1	3.216
3	-1	1	-1	2.620
4	1	1	-1	3.486
5	-1	-1	1	2.896
6	1	-1	1	3.714
7	-1	1	1	3.196
8	1	1	1	3.892
9	-α	0	0	2.348
10	α	0	0	3.570
11	0	- α	0	3.354
12	0	α	0	3.650
13	0	0	- α	3.232
14	0	0	α	3.546
15	0	0	0	3.846
16	0	0	0	3.684
17	0	0	0	3.610
18	0	0	0	3.794
19	0	0	0	3.810
20	0	0	0	3.728

^a^X_1_, maltose; X_2_, yeast extract; X_3_, soytone.

**Table 6:** Analysis of variance of the response surface quadratic model.

Source	DF^a^	Adj SS^b^	Adj MS^c^	*F*-value	*P*-value
Model	9	3.99061	0.44340	33.22	< 0.0001
X_1_	1	1.95502	1.95502	146.46	< 0.0001
X_2_	1	0.13981	0.13981	10.47	0.0089
X_3_	1	0.42885	0.42885	32.13	0.0002
X_1_X_2_	1	0.00002	0.00002	0.00	0.9714
X_1_X_3_	1	0.00088	0.00088	0.07	0.8024
X_2_X_3_	1	0.00065	0.00065	0.05	0.8301
X_1_^2^	1	1.23057	1.23057	92.18	< 0.0001
X_2_^2^	1	0.14479	0.14479	10.85	0.0081
X_3_^2^	1	0.28321	0.28321	21.22	< 0.0010
Residual	10	0.13349	0.01335		
Lack of fit	5	0.09443	0.01889	2.42	0.1774
Pure error	5	0.03906	0.00781		
Total	19	4.12410			

R^2^ = 0.9676; adjusted R^2^ = 0.9385; R = 0.9837.

^a^Degree of freedom.

^b^Sum of squares.

^c^Mean square.
